# A case of lung adenocarcinoma harboring *EGFR* mutation and *EML4-ALK* fusion gene

**DOI:** 10.1186/1471-2407-12-558

**Published:** 2012-11-26

**Authors:** Hisashi Tanaka, Akihito Hayashi, Takeshi Morimoto, Kageaki Taima, Yoshihito Tanaka, Michiko Shimada, Akira Kurose, Shingo Takanashi, Ken Okumura

**Affiliations:** 1 Hirosaki University Graduate School of Medicine, Course of Medical Sciences, Cardiology, Respiratory Medicine and Nephrology, Zaifu-cho 5, Hirosaki, 036-8562, Japan; 2 Department of Diagnostic Pathology, Hirosaki University Graduate School of Medicine, Hirosaki, Japan

**Keywords:** Lung cancer, *EGFR* mutation, EML4-ALK, Erlotinib

## Abstract

**Background:**

Lung cancer is the leading cause of cancer-related death worldwide. Epidermal growth factor receptor (EGFR) - tyrosine kinase inhibitor (TKI) is used for the patients with *EGFR*-mutant lung cancer. Recently, phase III studies in the patients with *EGFR*-mutant demonstrated that EGFR-TKI monotherapy improved progression-free survival compared with platinum-doublet chemotherapy. The echinoderm microtubule-associated protein-like 4 (*EML4*) *-* anaplastic lymphoma kinase (*ALK*) fusion oncogene represents one of the newest molecular targets in non-small cell lung cancer (NSCLC). Patients who harbor *EML4-ALK* fusions have been associated with a lack of *EGFR* or *KRAS* mutations.

**Case presentation:**

We report a 39-year-old patient diagnosed as adenocarcinoma harboring *EGFR* mutation and *EML4-ALK* fusion gene. We treated this patient with erlotinib as the third line therapy, but no clinical benefit was obtained.

**Conclusion:**

We experienced a rare case with EGFR mutation and *EML4-ALK*. Any clinical benefit using EGFR-TKI was not obtained in our case. The therapeutic choice for the patients with more than one driver mutations is unclear. We needs further understanding of the lung cancer molecular biology and the biomarker infomation.

## Background

Lung cancer is the leading cause of cancer-related death worldwide. Recent studies on personalized treatment by selecting patients who are likely to respond to a particular therapeutic agent may allow improved treatment efficacy. Patients with non-small cell lung cancer (NSCLC) harboring mutations in the epidermal growth factor receptor (*EGFR*) gene have dramatic response to the EGFR- tyrosine kinase inhibitor (EGFR-TKI) [1,2]. In 2007, the fusion of the anaplastic lymphoma kinase (*ALK*) with the echinoderm microtubule-associated protein-like 4 (*EML4*) was identified in NSCLC. *EML4-ALK* fusion gene arise as a result of an inversion in chromosome 2 that juxtaposed the 5 end of the *EML4* gene with the 3 end of the *ALK* gene. The frequency of the fusion gene is approximately 6.7% in NSCLC [3]. The clinical features of lung cancer that harbors *EML4-ALK* include light- or never-smokers, younger age, adenocarcinomas with acinar pattern or signet ring adenocarcinoma, and a lack of *EGFR* or *KRAS* mutations [4]. Patients who have both mutations are extremely rare.

## Case presentation

**Figure 1:**
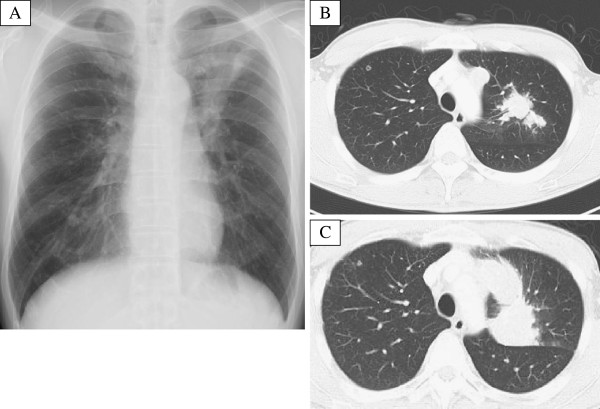
**A. Chest radiography revealed a mass shadow in the left upper lung field.** Figure 1**B**, 1**C**. Compared with the initial computed tomography (CT) of thorax **(B)** and the repeated CT at 30 days after erlotinib treatment **(C)**.

**Figure 2:**
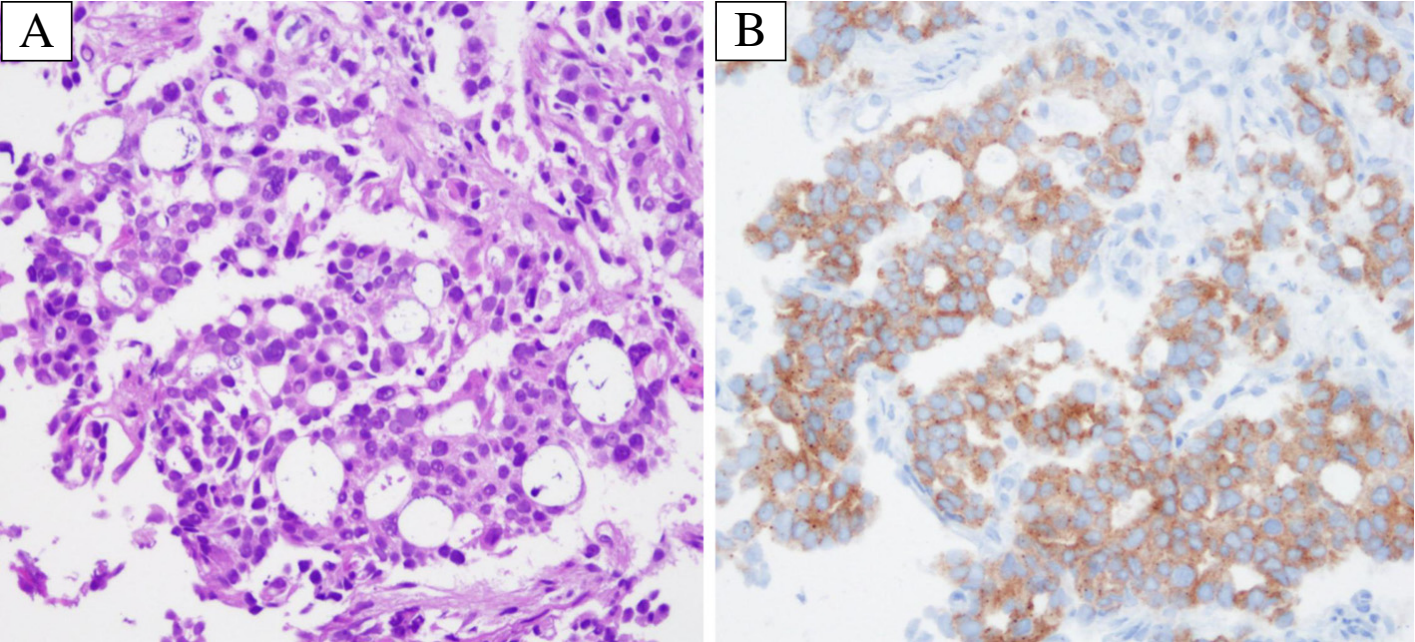
**Histology of the primary tumor: (A) well differentiated adenocarcinoma component with acinar pattern (HE ×200).(B)** Immunohistochemical examination revealed that tumor cells were positive for monoclonal anti-ALK antibody (5A4) (×200).

**Figure 3:**
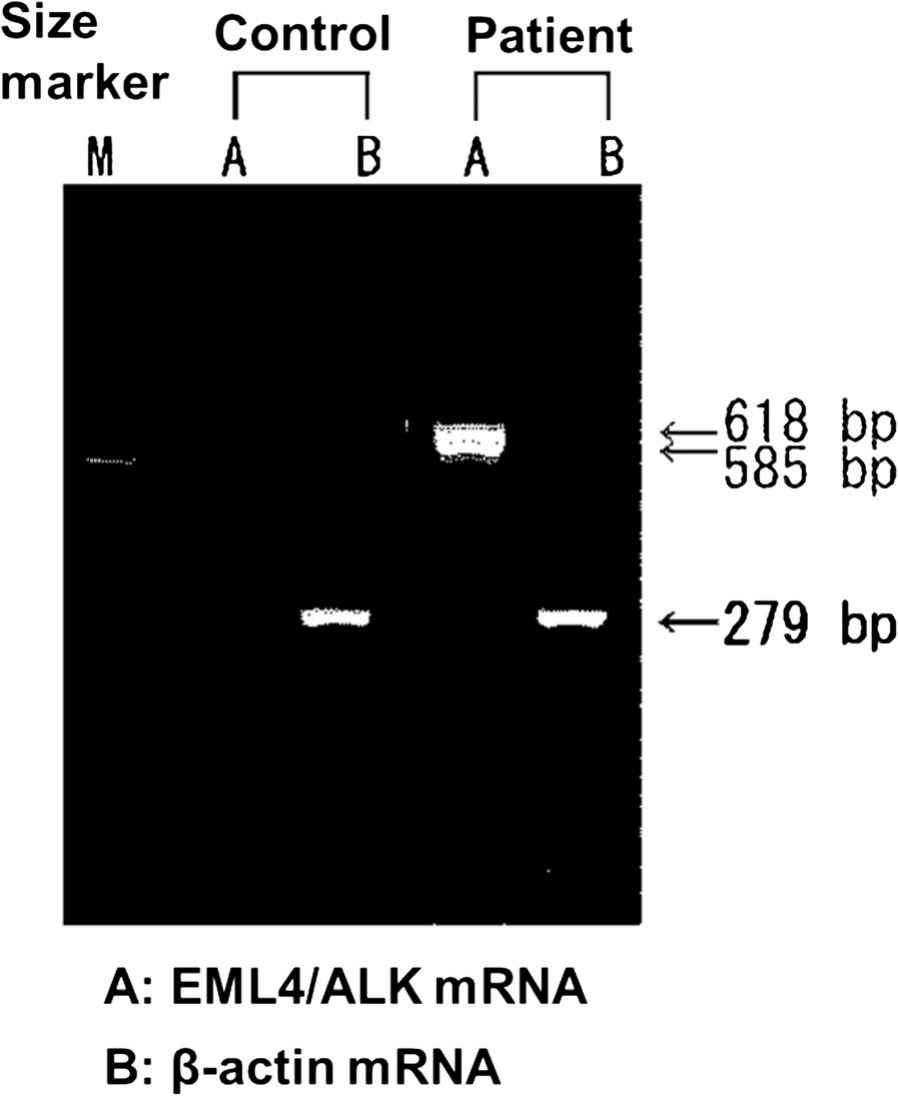
EGFR-gene mutation (L858R point mutation) was showed positive by Cycleave-PCR method.

**Figure 4:**
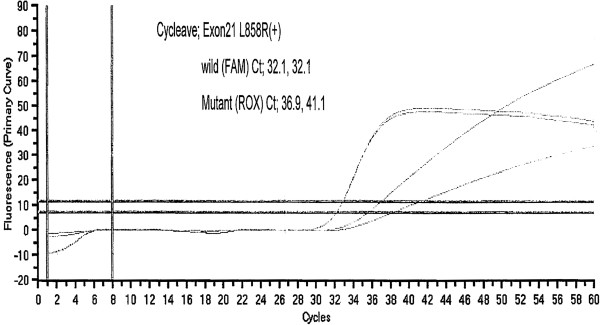
ALK fusion expression was showed positive by RT-PCR validation.

A 39-year-old man who is a light-smoker was referred to our hospital in June 2009 because of an abnormal shadow in the left upper field on chest X-ray (Figure 1A). Physical examination revealed no significant abnormalities. Computed tomography (CT) of the chest revealed a 40 mm tumor in the left S1+2 with multiple lung and bone metastases (cT4N3M1b). We conducted trans-bronchial lung biopsy (TBLB). The pathological diagnosis of the TBLB specimen was acinar adenocarcinoma (Figure 2A). In immunohistochemistry (IHC) staining, transcription factor-1 protein was positive. Laboratory findings were within normal range, except for the carcinoembryonic antigen (CEA) level of 4.7 ng/mL (normal range, 0 to 4.3 ng/mL) in the serum. We made a diagnosis of lung adenocarcinoma and treated the patient with the first line chemotherapy including cisplatin (80 mg/m^2^) and docetaxel (60 mg/m^2^) every three weeks up to three cycles. However, no remarkable response was observed. Therefore, the second line chemotherapy was conducted by pemetrexed (PEM) (500 mg/m^2^) therapy. PEM therapy was effective, and fifteen courses of PEM were administered to the patient. Thereafter new bone metastatic lesions were appeared. The patient had progressive disease (PD). We conducted re-biopsy to the patient to check *EGFR* mutation analysis by cycleave polymerase chain reaction technique (cycleave-PCR). L858R point mutation (in which leucine at amino acid 858 is replaced by arginine) was detected in the tumor cell (Figure 3). Therefore we treated the patient with erlotinib (150mg) therapy once a day. However, he had disease progression after 30 days (Figure 1B, C). We conducted the second re-biopsy to examine whether the patient had *ALK* fusion gene. *ALK* fusion gene was detected by reverse transcription polymerase chain reaction (RT-PCR) method (Figure 4). Additionally, IHC assay using a mouse monoclonal antibody for ALK antibody (Novocastra, Clone 5A4) revealed positive staining (Figure 2B). ALK inhibitor could not be used to the patient because ALK inhibitor was not approved in Japan at that time. The patient was treated fourth line chemotherapy three cycles, the other metastases emerged in his liver, and performance status became 3, therefore, palliative care was administered to the patient in December 2011.

## Discussion

We experienced a rare case of the patient who had both EGFR mutation and *EML4-ALK* fusion gene. To the best of our knowledge, five patients with both mutations have been reported so far in the world [5-8]. Four patients received EGFR-TKI therapy (Table 1). Two cases showed good response [5,6], whereas the other two cases did not [7]. We report the 5th case which also did not show good response. In general, the response rate to EGFR-TKI therapy in the patients with *EGFR* mutation is 70-80%, however, these 5 cases with both mutations tend to be less responsive. In the preclinical study, *EML4-ALK* positive NSCLC was not responsive to erlotinib therapy [9]. EGFR-TKI therapy showed no effects to the all 10 patients with *EML4-ALK* fusion gene [4], although, there were no patients harboring both *EGFR* mutation and *EML4-ALK* in these papers. Whereas, *EML4-ALK* positive patients had a longer progression free survival after PEM therapy compared with *EGFR* mutant patients [10].

**Table 1:** Patients characteristics and treatment outcomes by EGFR-TKI

**Citation**	**Age**	**Sex**	**Smoking history**	**Histology**	**EGFR mutation**	**EGFR-TKI response**	**ALK variant**
Kuo YW, et al.	72	Female	Never	Ad	Exon19 deletion	PR	Variant 1
Potat S, et al.	65	Female	Never	Ad	Exon19 deletion	CR	Unknown
Tiseo M, et al.	48	Male	Never	Adsq	Exon19 deletion	PD	Unknown
Zhang X, et al.	Unknown	Female	Never	Ad	Exon19 deletion	NA	Variant 3b
Present case	39	Male	Former	Ad	L858R	PD	Variant 3b

Abbreviations.

*Ad*: adenocarcinoma, *Adsq*: adeno-squamous carcinoma, *CR*: complete response.

*PR*: partial response, *PD*: progressive disease, *NA*: not evaluated.

In our case, the characteristics of the patient were young age, light-smoker and acinar pattern adenocaricinoma which showed similarity with the ones of EML4-ALK positive NSCLC. Additionally, PEM therapy showed a good response to our patient, whereas erlotinib therapy did not. In the cases with these both mutations, *EML4-ALK* gene may play a main role in the oncogenesis for some unknown reasons. Although ALK inhibitor was effective to *EML4-ALK* positive NSCLC [11], it was not on the market in Japan at that point. Further experience and the understanding of the lung cancer molecular biology are required for the better treatment of the cases with both EGFR mutation and *EML4-ALK* fusion gene.

## Conclusion

We report a rare case of lung cancer harboring both *EGFR* mutation and *EML4-ALK* fusion gene. PEM therapy showed a good response to the patient, whereas erlotinib therapy did not. Oncologists should be aware of the possibility of the multiple mutations.

## Consent

Written informed consent was obtained from the patient for publication of this case report and accompanying images. A copy of the written consent is available for review by the Editor-in-Chief of this journal.

## Abbreviations

EGFR: Epidermal growth factor receptor; TKI: Tyrosine kinase inhibitor; EML4: Echinoderm microtubule-associated protein-like 4; ALK: Anaplastic lymphoma kinase; NSCLC: Non-small cell lung cancer; TBLB: Trans-bronchial lung biopsy; CT: Computed tomography; IHC: Immunohistochemistry; CEA: Carcinoembryonic antigen; PEM: Pemetrexed; PD: Progressive disease; Cycleave-PCR: Cycleave polymerase chain reaction technique; RT-PCR: Reverse transcription polymerase chain reaction.

## Competing interests

The authors declare that they have no competing interests.

## Authors' contributions

HT and AH prepared the manuscript and the literature search; KT reviewed and edited the manuscript; TM and MS corrected and revised the manuscript; YT treated and observed the patient; AK performed the histopathological, immunohistochemical examinations; and ST and KO reviewed the manuscript. All authors read and approved the final manuscript.

## Pre-publication history

The pre-publication history for this paper can be accessed here:

http://www.biomedcentral.com/1471-2407/12/558/prepub
